# Supplementary figures and images for: Development and characterization of rabbit monoclonal antibodies that recognize human spermine oxidase and application to immunohistochemistry of human cancer tissues
Source: PLoS One. 2022 Apr 22;17(4):e0267046. doi: 10.1371/journal.pone.0267046 (PMC9032377; doi:10.1371/journal.pone.0267046)

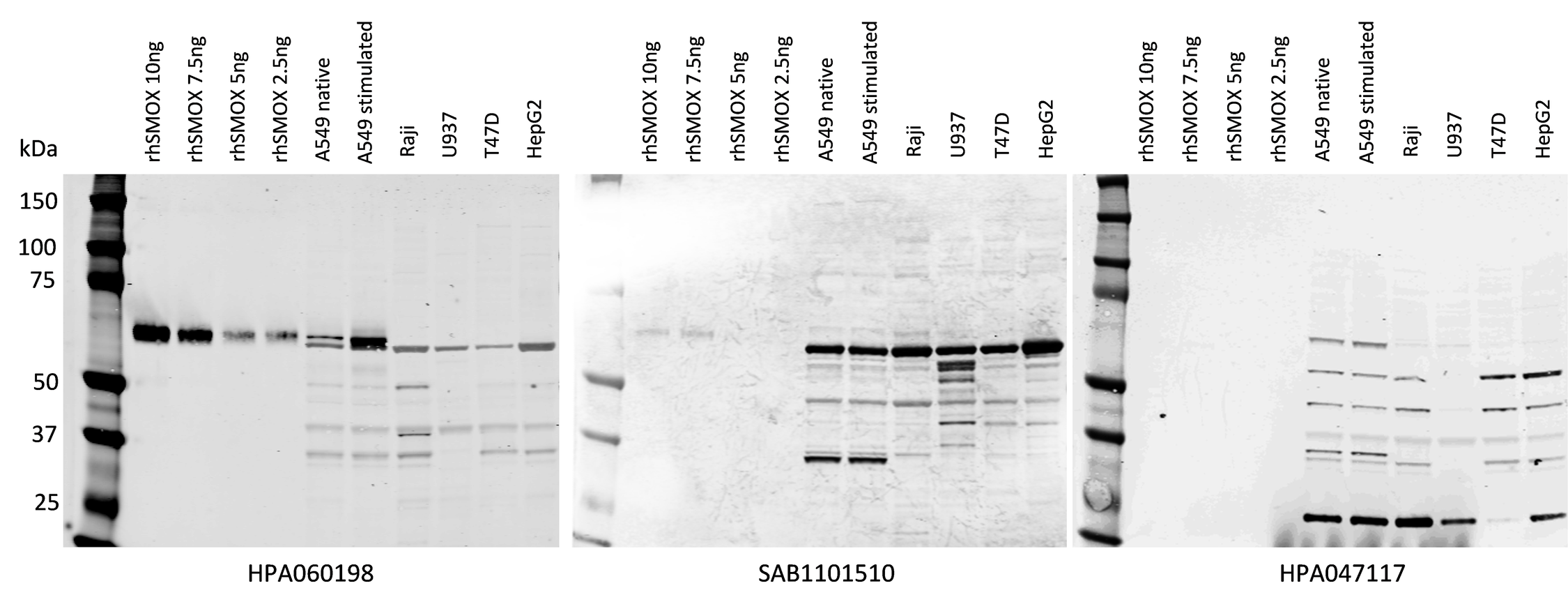

Supplement: S1 Fig — Western blot using commercial polyclonal antibodies probing their capacity to recognize rhSMOX and native hSMOX in different cell line extracts. (TIF) [file pone.0267046.s001.tif]

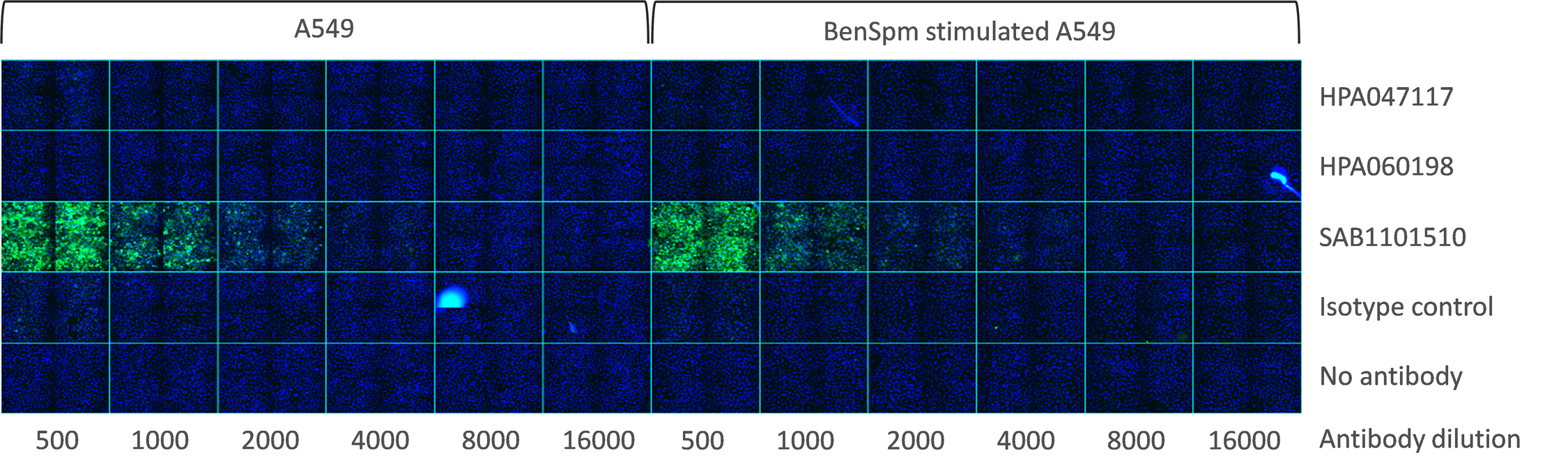

Supplement: S2 Fig — Immunofluorescence of A549 cells and BENSpm stimulated A549 cells using three different commercial polyclonal antibody preparations at various dilutions. Nuclei (DAPI) are shown in blue, and hSMOX (α-Rabbit-IgG-AF488) is shown in green. (TIF) [file pone.0267046.s002.tif]

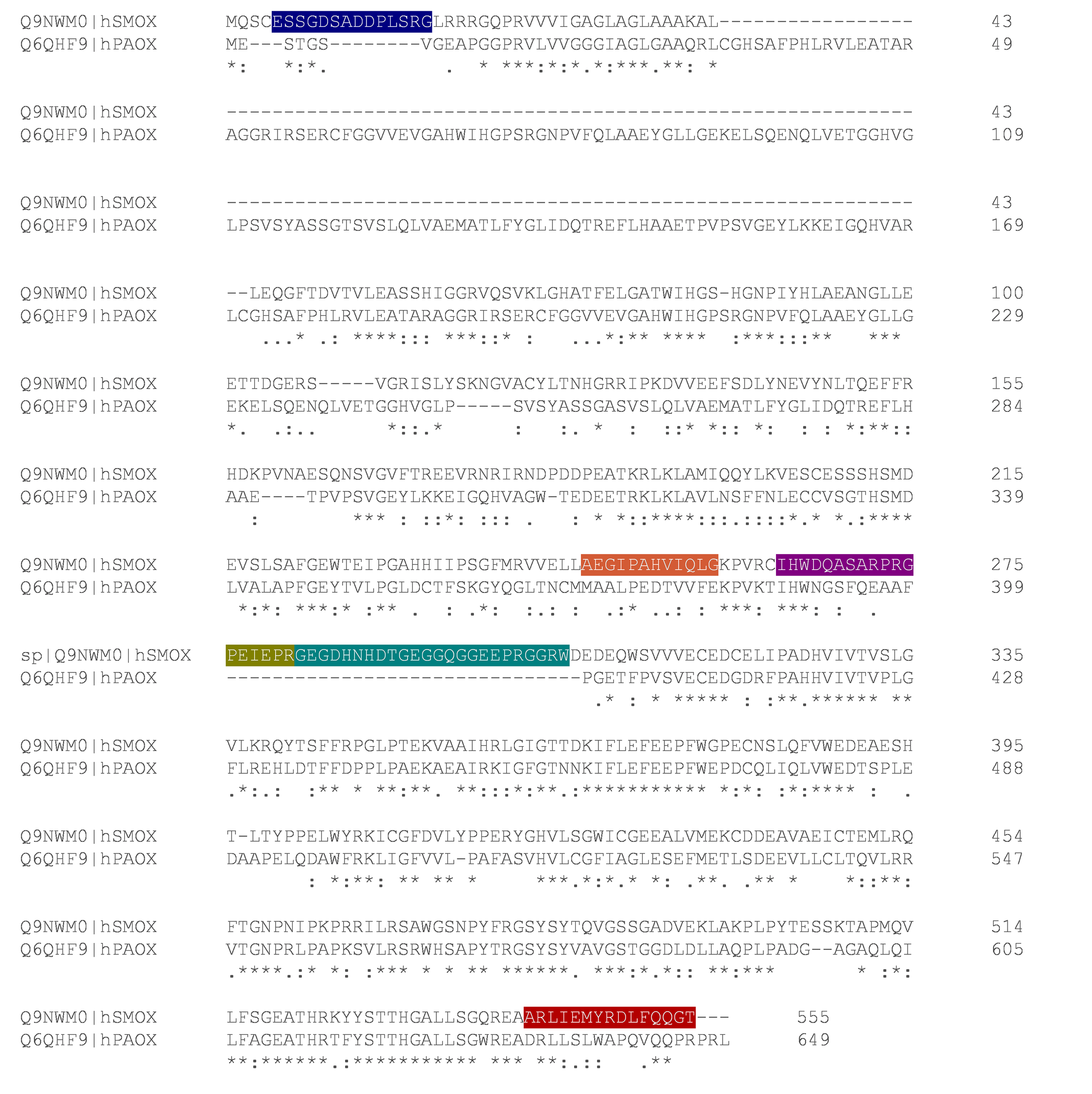

Supplement: S3 Fig — The peptide sequences used for immunization are highlighted using the color coding of Fig 1 in the main text (NT: blue; SL: orange; LL2: purple; LL1 and LL2 overlap: yellow; LL1: cyan; CT: red). (TIF) [file pone.0267046.s003.tif]

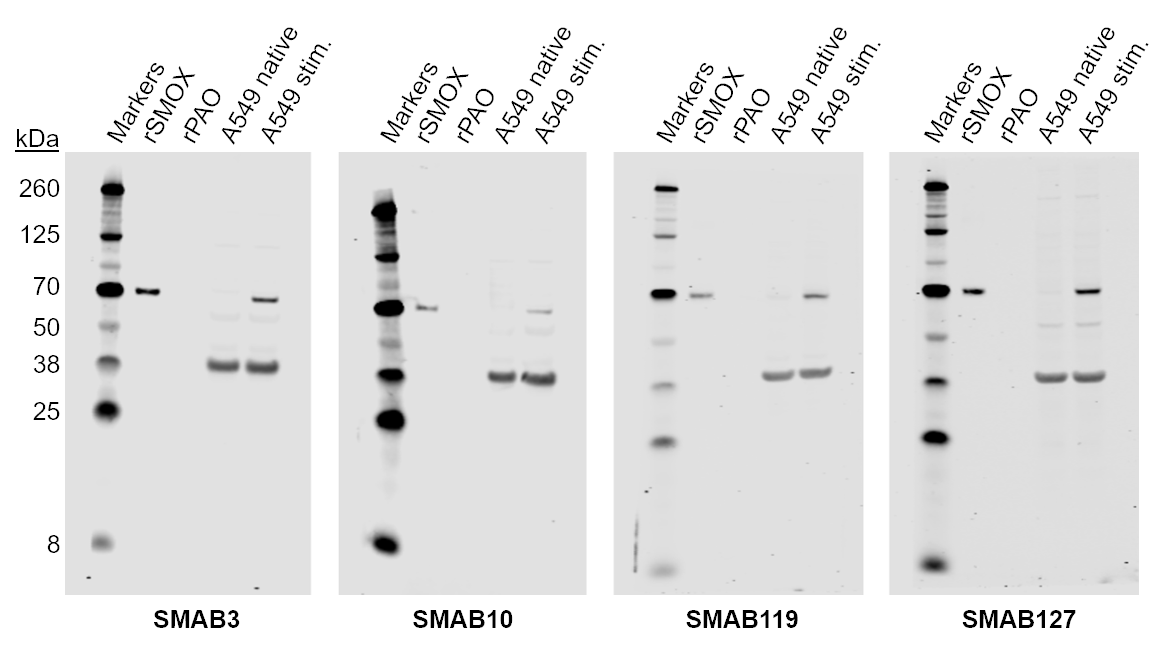

Supplement: S4 Fig — Western blots are overlaid with GAPDH loading control. (TIF) [file pone.0267046.s004.tif]

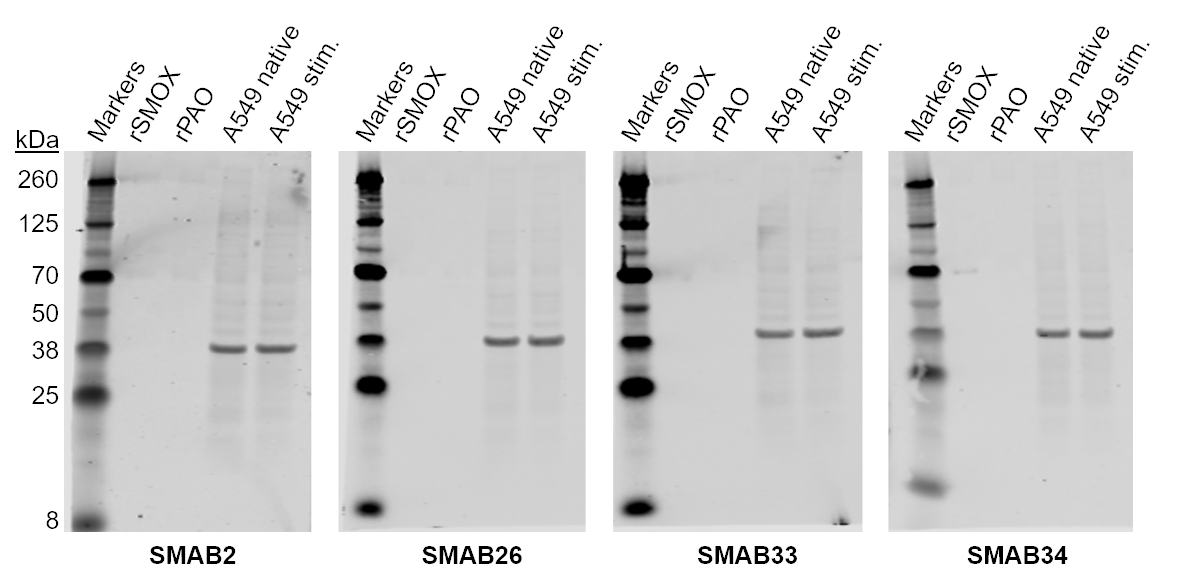

Supplement: S5 Fig — Faint bands visible near 70 kDa marker, nonspecific bands are similarly stained. GAPDH was used as a loading control (see S4 Fig). Loading: 10 ug lysate, 1 ng rhSMOX, 1 ng rhPAO. (TIF) [file pone.0267046.s005.tif]

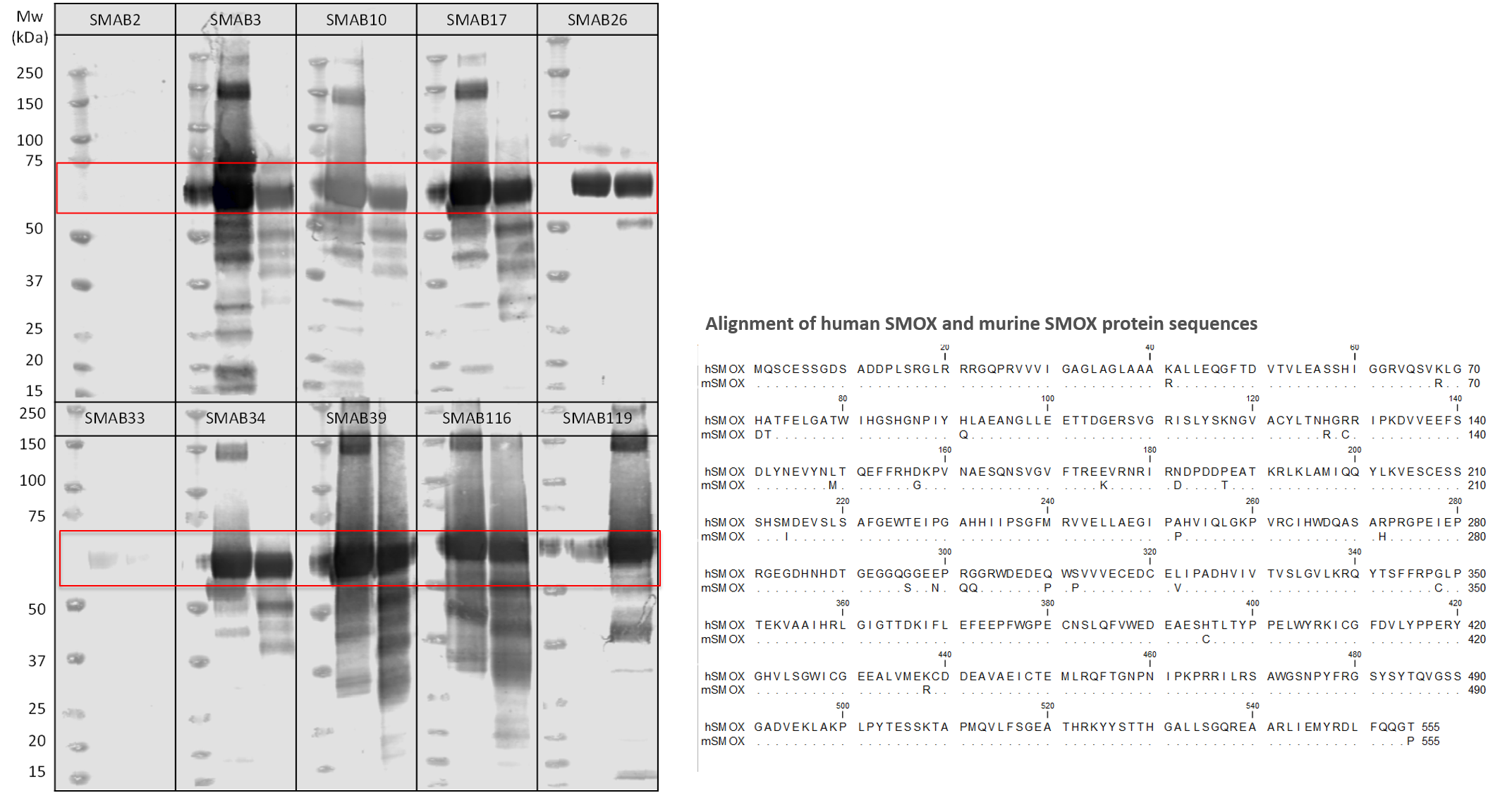

Supplement: S6 Fig — Western blot assessing the reactivity of selected SMABs towards recombinantly expressed human and murine SMOX (1ug SMOX per lane; 1:1000 dilution of primary detection SMAB). For all samples, the order is: Lane 1 Marker, lane 2 Human SMOX, lane 3 Murine SMOX. (TIF) [file pone.0267046.s006.tif]

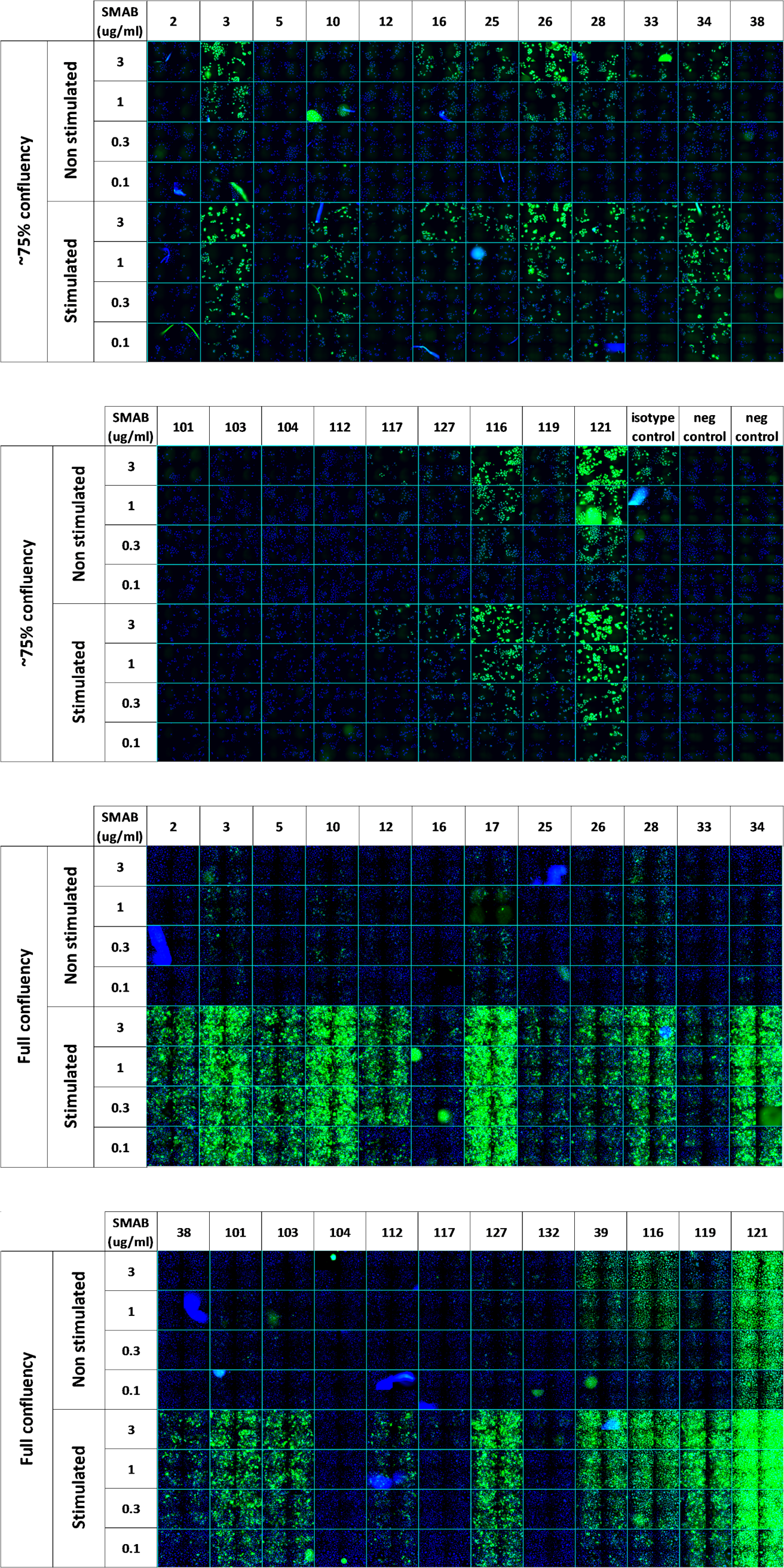

Supplement: S7 Fig — Immunofluorescence of fixed non-stimulated and BENSpm stimulated A549 cells at ~75% and full cell confluency using different SMABs at various concentrations. Nuclei (DAPI) are shown in blue, and SMAB (α-Rabbit-IgG-AF488) is shown in green. (TIF) [file pone.0267046.s007.tif]

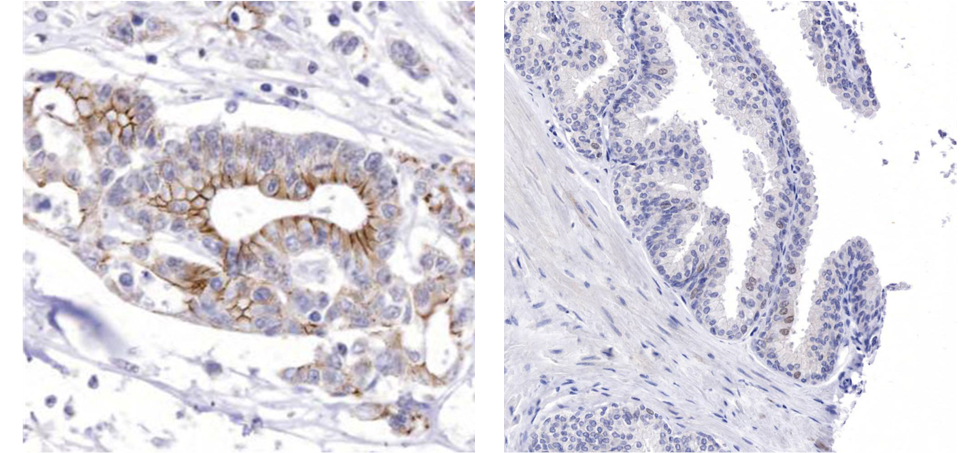

Supplement: S8 Fig — Image illustrating apical membranous staining observed in colon cancer (left) and nuclear staining in normal prostate tissue (right). (TIF) [file pone.0267046.s008.tif]
