# Supplementary material for: Development and characterization of rabbit monoclonal antibodies that recognize human spermine oxidase and application to immunohistochemistry of human cancer tissues
Source: PLoS One. 2022 Apr 22;17(4):e0267046. doi: 10.1371/journal.pone.0267046 (PMC9032377; doi:10.1371/journal.pone.0267046)

WB gels used for generating Table 1

Blots were imaged with the LI-COR Odyssey (LI-COR Biosciences, Lincoln, NE)

SMO=human recombinant SMOX

6970= catalytically active recombinant rhSMOXΔLL mutant that does not contain the LL1 and LL2 sequences (Δ286-317)

hPAO=huma recombinant PAOX

A^+= BENSpm stimulated A549 cells

A^-= non stimulated A549 cells

Raji= Raji cells used as negative control

SMAB2 Low exposure | SMAB3 | SMAB2 High exposure | SMAB3

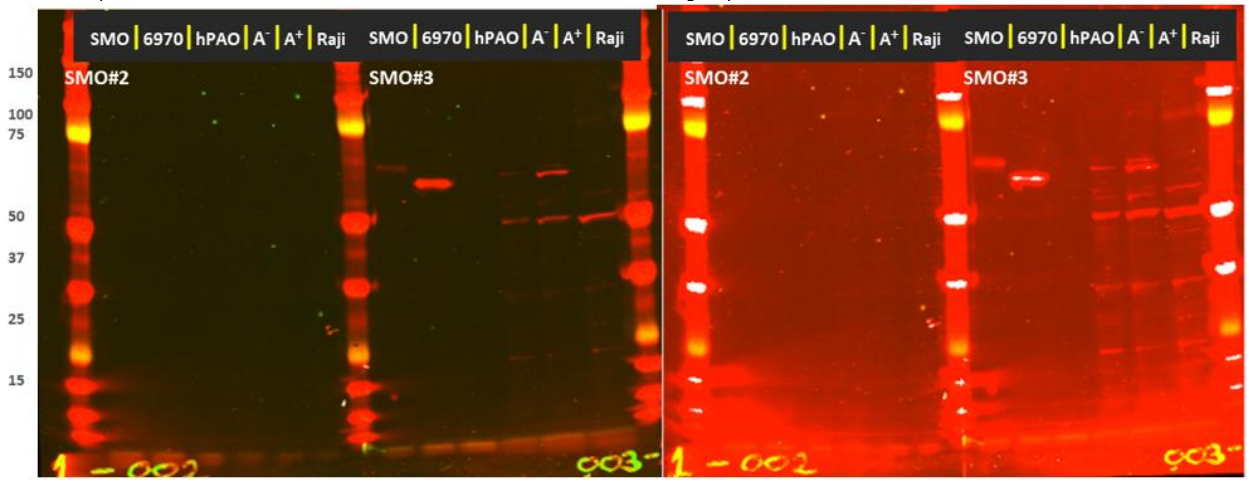

SMAB12 SMAB16 SMAB12 SMAB16

Low exposure High exposure

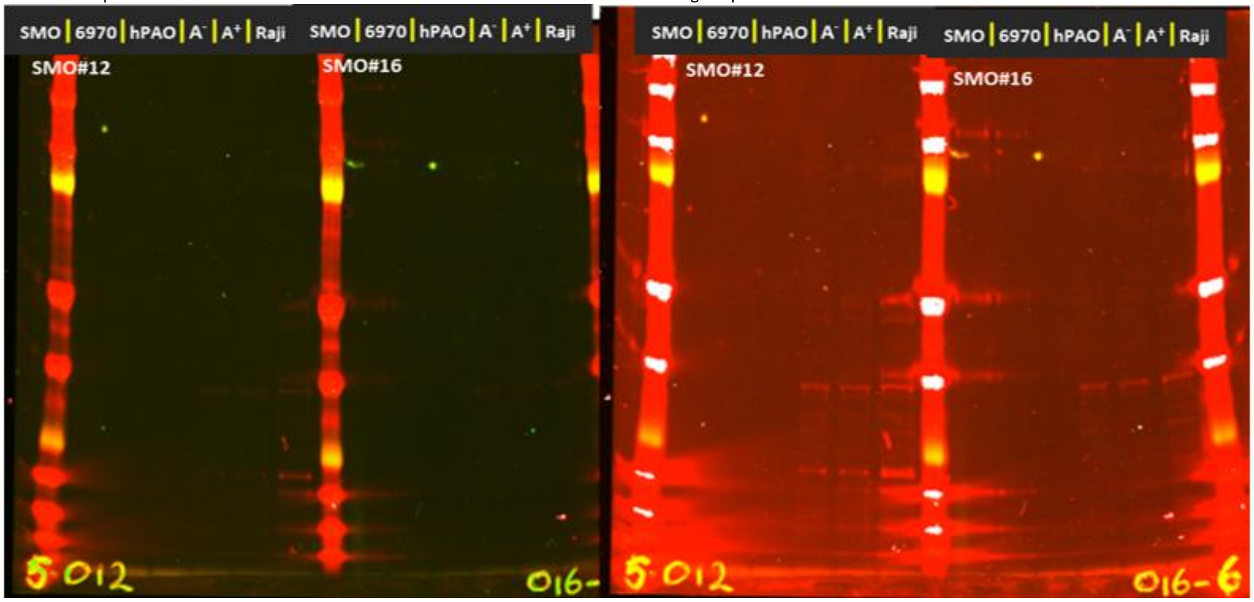

SMAB25 SMAB26 SMAB25 SMAB26

Low exposure High exposure

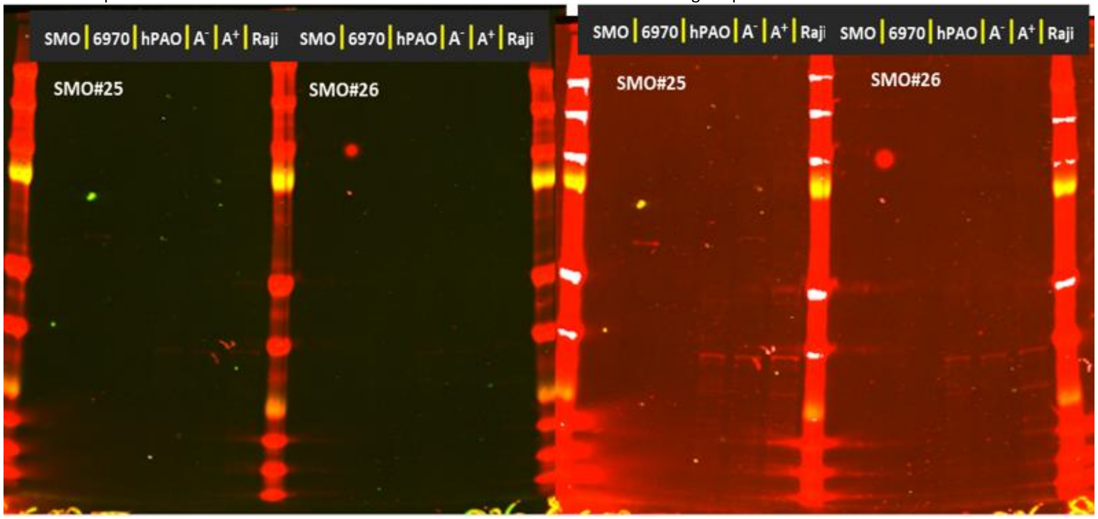

SMAB28 SMAB33

Low exposure

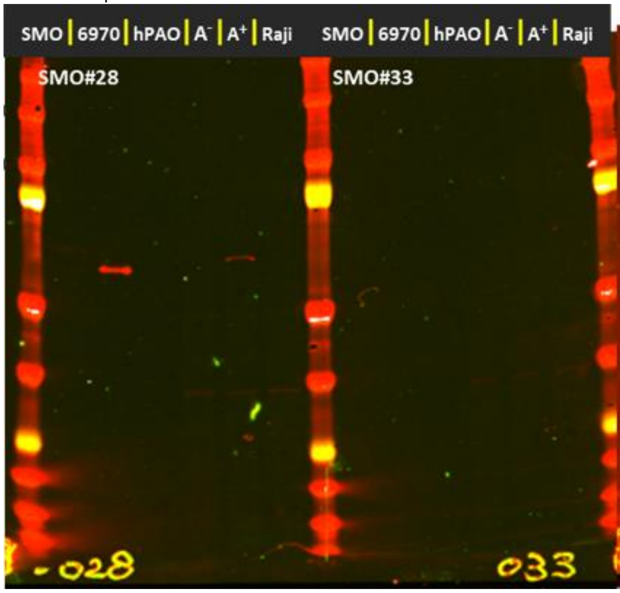

SMAB28 SMAB33

High exposure

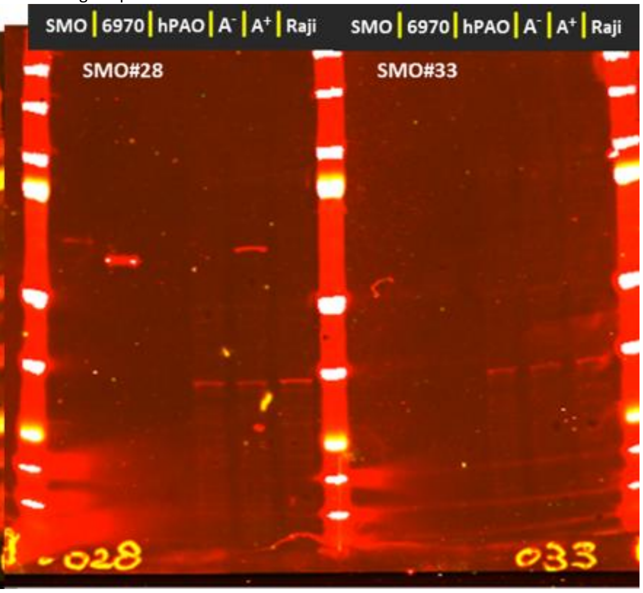

SMAB101 SMAB102

Low exposure

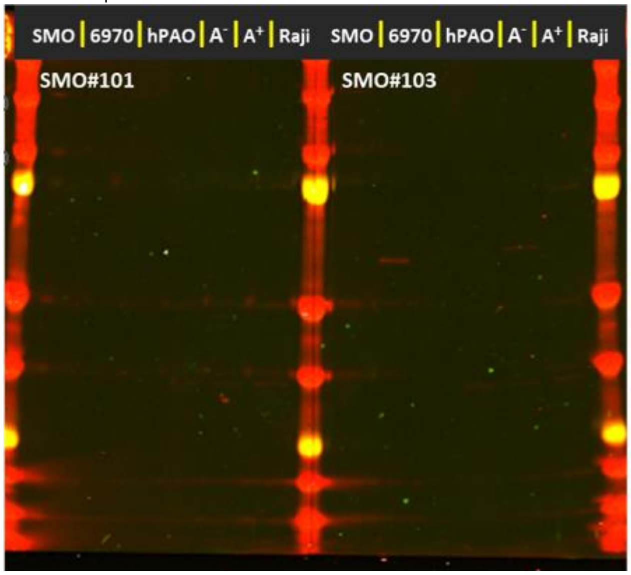

SMAB101 SMAB103

High exposure

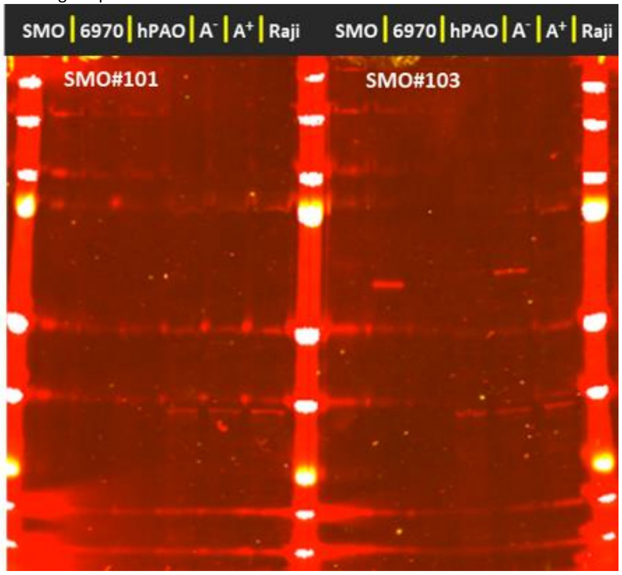

SMAB34 SMAB38 SMAB34 SMAB38

Low exposure High exposure

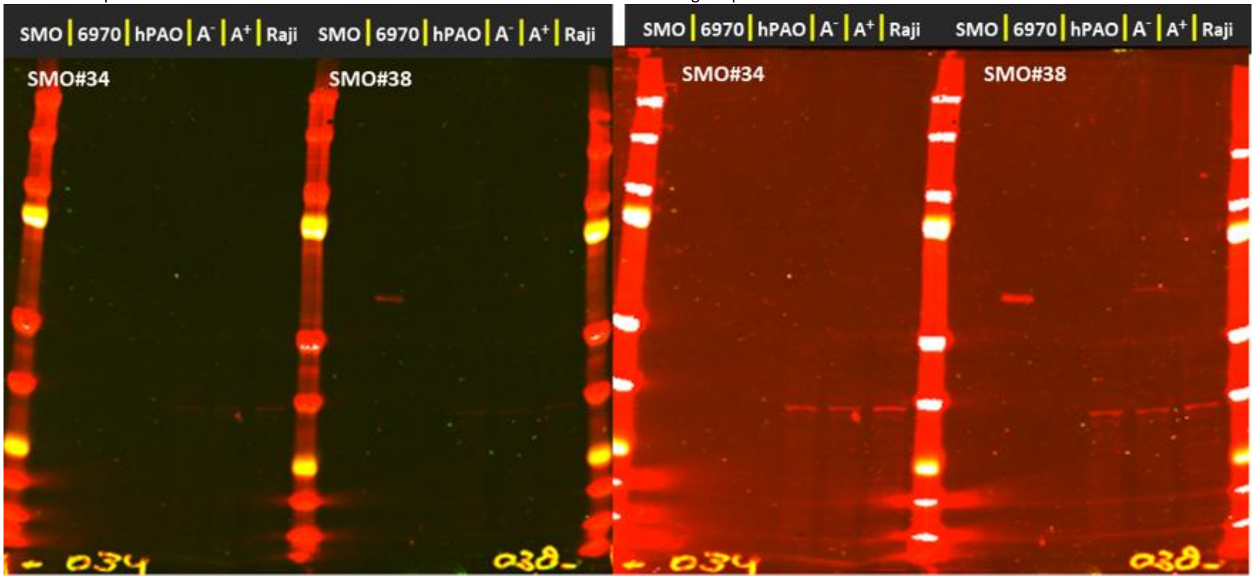

SMAB104 SMAB112 SMAB104 SMAB112

Low exposure High exposure

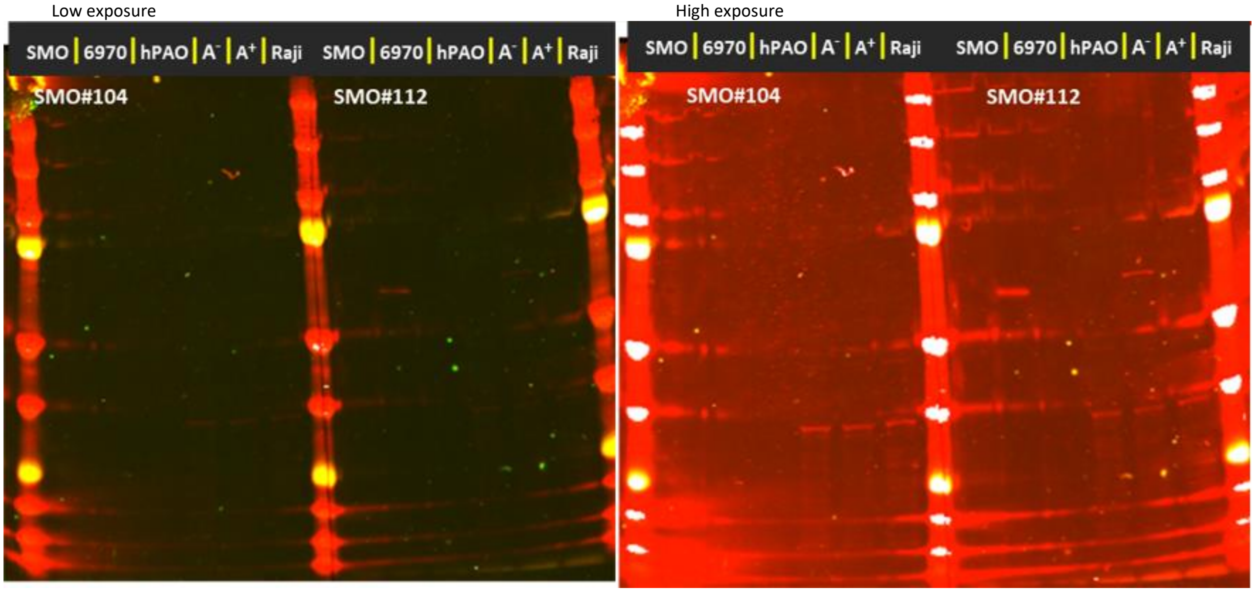

SMAB117 SMAB127

Low exposure

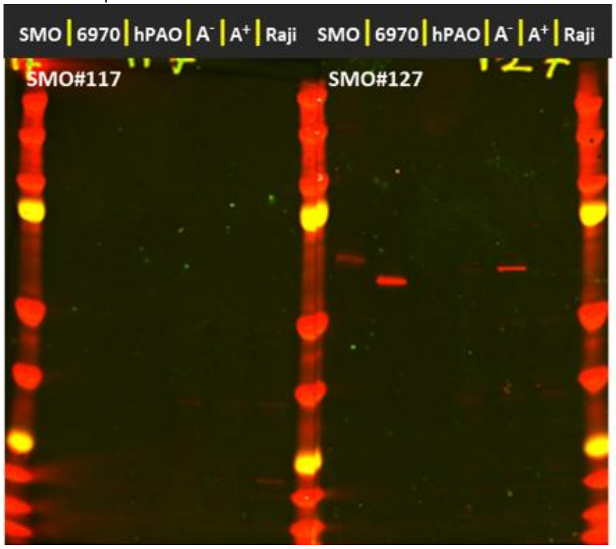

SMAB117 SMAB127

High exposure

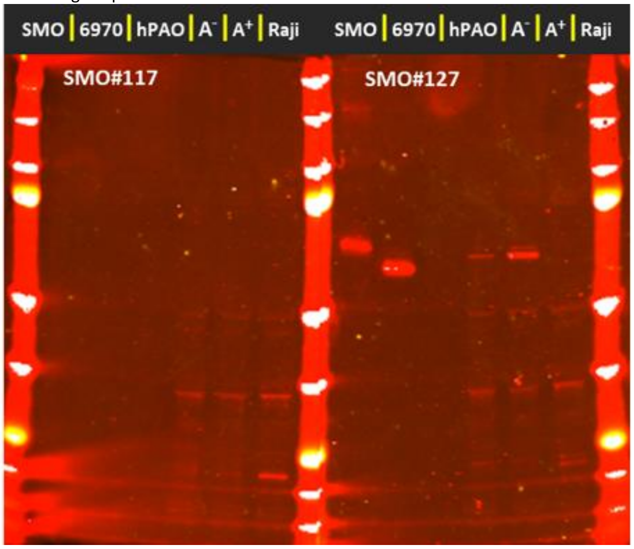

SMAB132 SMAB132

Low exposure High exposure

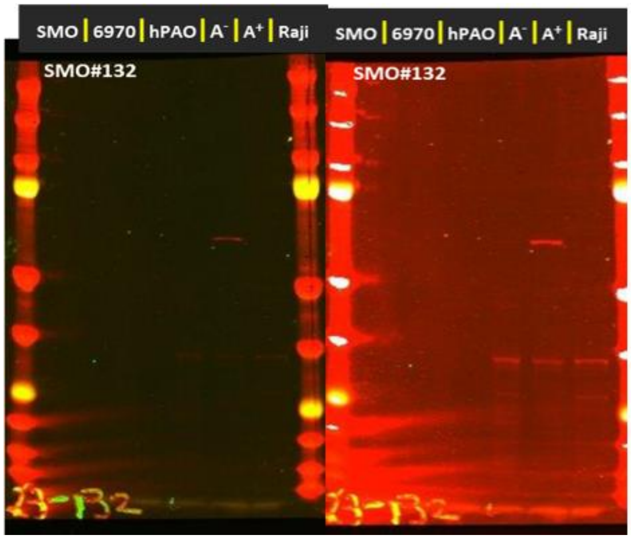

SMAB17 SMAB17

Low exposure High exposure

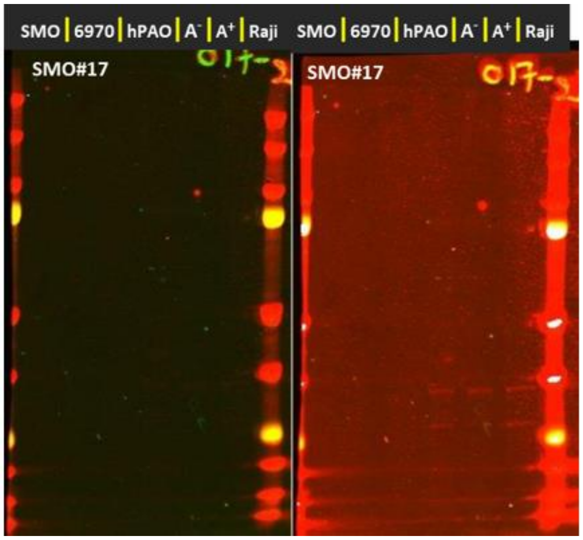

WB gels used for generating figure 4 of the manuscript

Blots were imaged with the LI-COR Odyssey (LI-COR Biosciences, Lincoln, NE)

rSMOX=human recombinant SMOX

rhPAOX=huma recombinant PAOX

BENSpm= BENSpm stimulated A549 cells

Veh= non stimulated A549 cells

Raji= Rajicells used as negative control

SMAB119

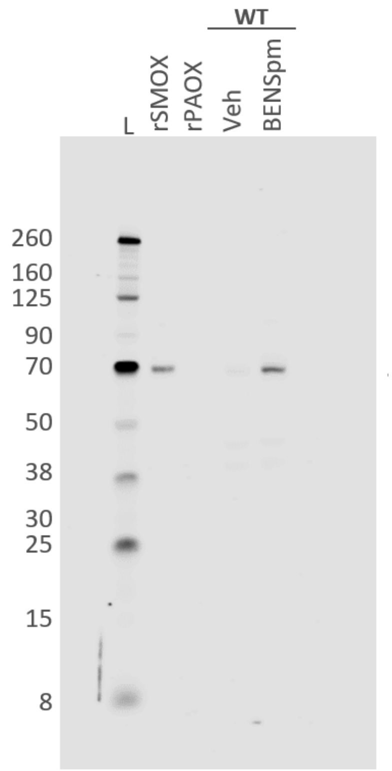

SMAB3

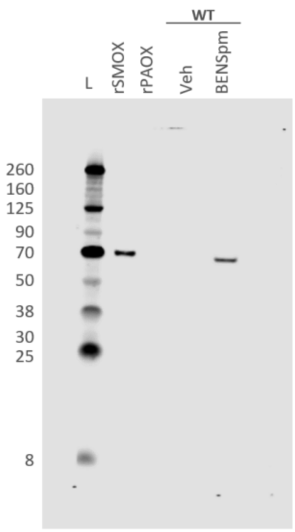

SMAB10

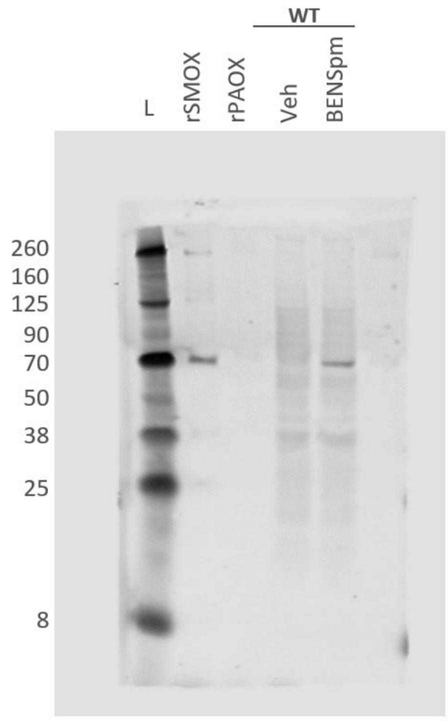

WB gels used for generating supplementary figure 5 of the manuscript

Blots were imaged with the LI-COR Odyssey (LI-COR Biosciences, Lincoln, NE)

rSMOX=human recombinant SMOX

rhPAOX=huma recombinant PAOX

BENSpm= BENSpm stimulated A549 cells

Veh= non stimulated A549 cells

Raji= Rajicells used as negative control

SMAB2

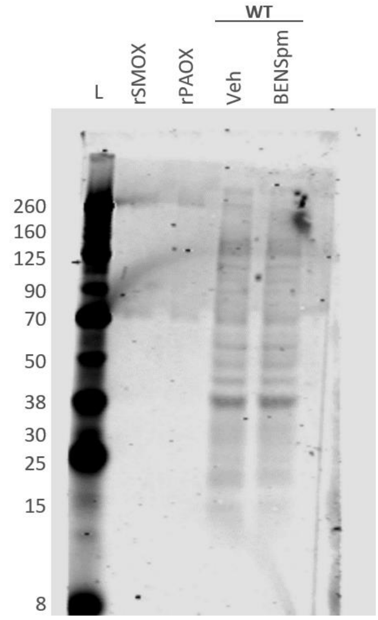

SMAB26

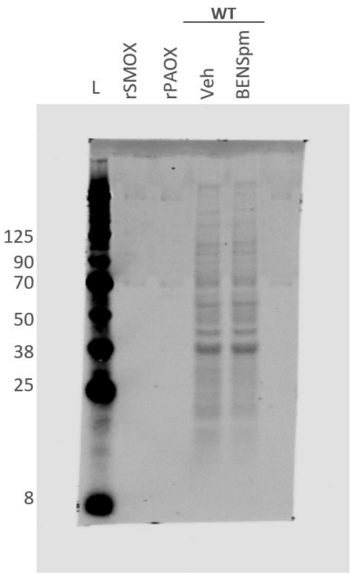

SMAB33

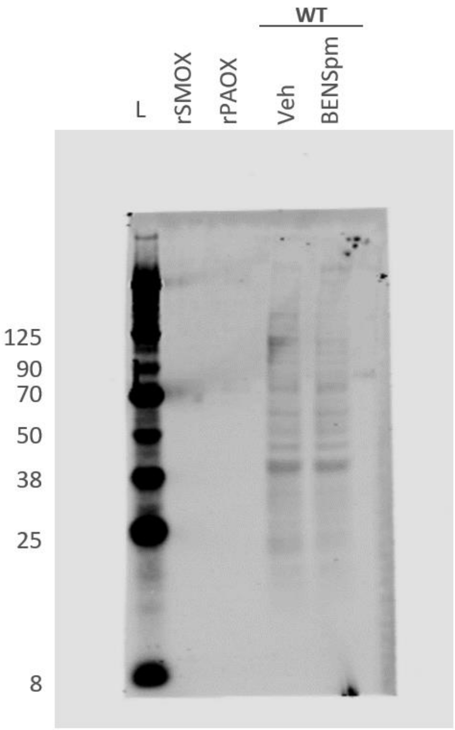

SMAB34

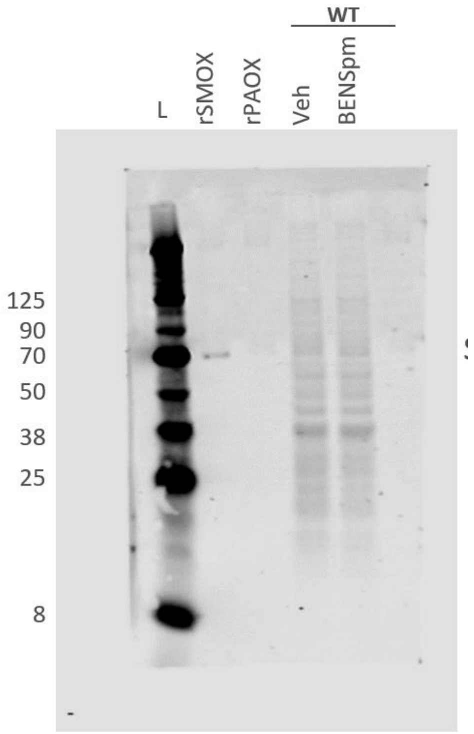

WB gels used for generating supplementary figure 6

Blots were imaged with the LI-COR Odyssey (LI-COR Biosciences, Lincoln, NE)

rhSMO=human recombinant SMOX

rmSMOX= maouse recombinant SMOX

Top gel:

Proteins loaded:

Marker|rhSMOX|rmSMOX

Primary antibody:

|SMAB2|SMAB3|SMAB10|SMAB17|SMAB26

Bottom gel:

Proteins loaded:

Marker|rhSMOX|rmSMOX

Primary antibody:

Marker|SMAB33|SMAB34|SMAB39|SMAB116|SMAB119

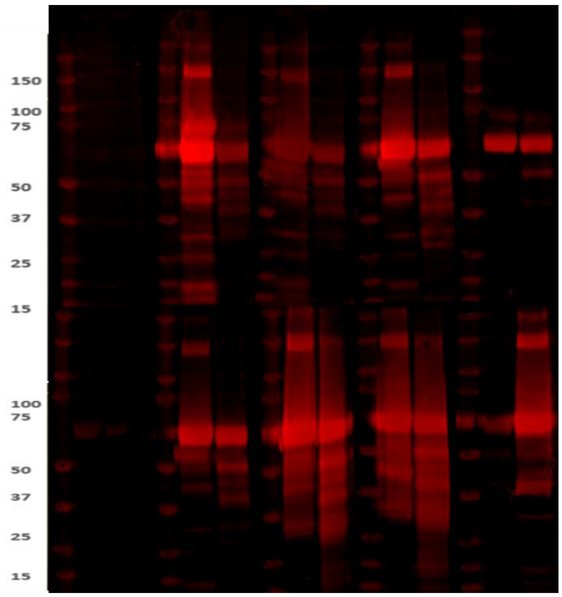

Supplement: S1 Raw images — (PDF) [file pone.0267046.s010.pdf]
