# Supplementary material for: Development and characterization of rabbit monoclonal antibodies that recognize human spermine oxidase and application to immunohistochemistry of human cancer tissues
Source: PLoS One. 2022 Apr 22;17(4):e0267046. doi: 10.1371/journal.pone.0267046 (PMC9032377; doi:10.1371/journal.pone.0267046)
Supplement: Attachment — Submitted filename: Tepper et al_Response to the reviewer.docx [file pone.0267046.s011.docx]

# Response to the reviewer of the manuscript:

**Development and characterization of rabbit monoclonal antibodies that recognize human spermine oxidase and application to immunohistochemistry of human cancer tissues**

Armand W.J.W. Tepper^1^; Gerald Chu^2^; Vincent N.A. Klaren^1^; Jay H. Kalin^2^; Patricia Molina-Ortiz^3^; Antonietta Impagliazzo^1,4*^

Reviewer #1 accomplished the benefit of your multiple monoclonal SMOX antibodies, but he invites the authors revising the list of references. The editor already invited the author to pose more attention on this point and ignoring it is certainly not a positive message for the editor and colleagues cited erroneously in the manuscript. Secondly, reviewer # 1 suggests to the authors providing evidences for a cross reaction with other model mammalian organisms beside human. It could not be an issue but it needs more experiments or comments.

- We apologize for the number of errors in the previous list of references. We have checked and revised the reference section and hopefully provided the correct author's names and format.
- Furthermore, we have reported the cross-reactivity of some of these antibodies with recombinant murine SMOX (Table 1 of the manuscript, line 389). We have added the figure of the WB in the supplementary information as well as an alignment of the human and murine sequence (line 382 and S6 Fig. line 384). We recognize that the quality of the blots is suboptimal, but unfortunately, we are unable to perform additional experiments due to the changes in circumstances. We hope, however, that this is convincing enough to illustrate the suitability of those mAbs for use in work with mouse models or tissues.

Reviewer #2 recognizes the work as solid with appropriate amount of experiments. Unfortunately, still the description of the statistical analysis is completing missing, as well the editor asked upon the original submission. Providing a paragraph in MM and mentioning the appropriate statistical analysis used in each experiment means which kind of statistical tool have been applied (student-T test, ANOVA, median range, etc). Moreover, all the Material and Methods section should be revised and described in more details (as an example, in immune-histochemistry, how have negative control been determined?).

- The statistical analysis is now clearly outlined in a paragraph added to the MM as the reviewer requested (row 253, Fig 5 legend at row 415). We have also added a S1 Table with the raw data and all the statistical parameters used in our analysis.
- Furthermore, additional detail has been added to the MM section regarding the AlphaLisa (row 156) and Immunohistochemistry (row 205-219; 223-233) with clarification on the negative control determination. In brief, we selected Raji cells as negative control because publicly available transcriptomic data indicate a low basal hSMOX expression in this cell line (row 278), consistent with our own data. Raji cells were used to produce paraffin-embedded cell and xenograft samples to serve as a negative control in IHC (Fig 7).
